# Supplementary material for: Targeting Retinaldehyde Dehydrogenases to Enhance Temozolomide Therapy in Glioblastoma
Source: Int J Mol Sci. 2024 Oct 26;25(21):11512. doi: 10.3390/ijms252111512 (PMC11546810; doi:10.3390/ijms252111512)
Supplement: Supplementary file 1 [file ijms-25-11512-s001.zip › ijms-3233163-supplementary.pdf]

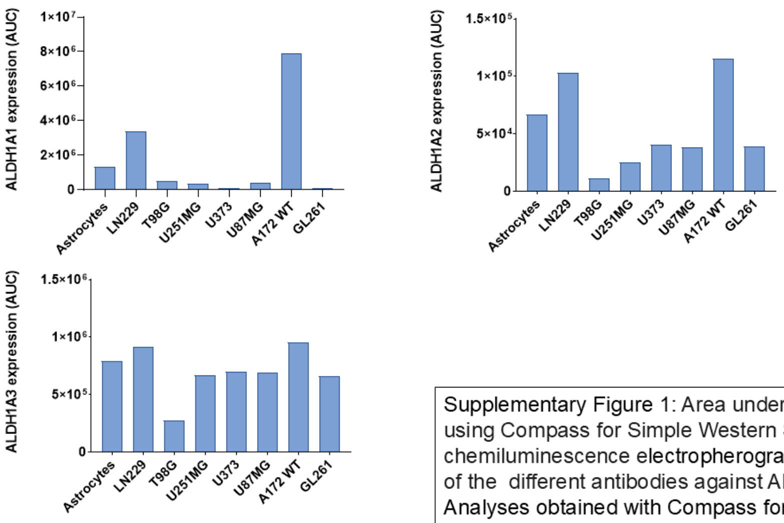

Supplementary Figure 1: Area under the curve (AUC) quantified using Compass for Simple Western Software from chemiluminescence electropherogram generated by the signals of the different antibodies against ALDH1A1, 1A2, 1A3. Analyses obtained with Compass for Simple Western Software (ProteinSimple, CA, USA). Electropherogram of ALDH1A1, 1A2 and 1A3 protein expression detected by a chemiluminescence readout on Wes lane view.

Figure S1
